# The Enfacement Illusion Is Not Affected by Negative Facial Expressions

**DOI:** 10.1371/journal.pone.0136273

**Published:** 2015-08-20

**Authors:** Brianna Beck, Flavia Cardini, Elisabetta Làdavas, Caterina Bertini

**Affiliations:** 1 Centro studi e ricerche in Neuroscienze Cognitive (CNC), University of Bologna, Cesena, Italy; 2 Department of Psychology, University of Bologna, Bologna, Italy; University of Udine, ITALY

## Abstract

Enfacement is an illusion wherein synchronous visual and tactile inputs update the mental representation of one’s own face to assimilate another person’s face. Emotional facial expressions, serving as communicative signals, may influence enfacement by increasing the observer’s motivation to understand the mental state of the expresser. Fearful expressions, in particular, might increase enfacement because they are valuable for adaptive behavior and more strongly represented in somatosensory cortex than other emotions. In the present study, a face was seen being touched at the same time as the participant’s own face. This face was either neutral, fearful, or angry. Anger was chosen as an emotional control condition for fear because it is similarly negative but induces less somatosensory resonance, and requires additional knowledge (i.e., contextual information and social contingencies) to effectively guide behavior. We hypothesized that seeing a fearful face (but not an angry one) would increase enfacement because of greater somatosensory resonance. Surprisingly, neither fearful nor angry expressions modulated the degree of enfacement relative to neutral expressions. Synchronous interpersonal visuo-tactile stimulation led to assimilation of the other’s face, but this assimilation was not modulated by facial expression processing. This finding suggests that dynamic, multisensory processes of self-face identification operate independently of facial expression processing.

## Introduction

The mental self-face representation comprises an important part of both self-identity and the mental representation of one’s own body [1]. Indeed, mirror self-recognition is often taken as a hallmark of self-consciousness in general [2,3]. Self-recognition tasks measuring processing of one’s own face compared to the faces of others have found that the self-face is more salient and processed more efficiently [4–6]. Additionally, imaging studies seeking the neural basis of visual self-recognition have implicated a fronto-parietal network, particularly in the right hemisphere, that includes a spatial representation of the self in the inferior parietal lobule and higher-order evaluations of the self versus others in anterior areas such as the inferior frontal gyrus, the anterior cingulate cortex, and the anterior insula (see [1] for a review).

While the studies described above focused on visual self-recognition, more recent accounts of self-recognition have attempted to explain how the mental self-representation, including the self-face, is constructed and updated over time via the convergence of multimodal inputs [7]. This line of research has revealed that multisensory information can update the self-face representation, and, under certain circumstances, may blur the distinction between self and other. Seeing another person’s face touched in synchrony with touch on one’s own face—a procedure called interpersonal multisensory stimulation (IMS)—results in an “enfacement” illusion [8–11]. Concurrent visual and tactile inputs update the mental representation of the self-face, causing the subjects to accept more of the other person’s facial features as their own [8,10]. This effect has been replicated using additional measures of self/other merging, including the distance the participant chooses between two circles representing “self” and “other” (a variant of the Inclusion of the Other in the Self scale [12]) and a questionnaire assessing the subjective experience of the enfacement illusion adapted from the rubber hand illusion questionnaire [9–11]. Moreover, an interpersonal visuo-tactile experience can influence feelings of affinity with another person. Synchronous IMS increases ratings of attraction to the other, biases inferences about the other’s personality towards one’s own personality traits, and makes one more influenced by the other’s estimate in a quantity estimation task [9]. A recent study also demonstrated that viewing a person from a different ethnic background being touched in synchrony with touch on one’s own face can improve somatosensory resonance with the outgroup member [13].

Being interpersonal by definition, enfacement might be influenced by the social and emotional context of the IMS experience. Supporting this notion, the strength of the enfacement illusion is positively correlated with participants’ empathic traits, specifically the tendencies to adopt another’s point of view and to share others’ emotions and feelings [10]. In addition, social and emotional dimensions of faces can influence self-face recognition in self/other morphs (without any multisensory stimulation). Consistent with an implicit self-enhancement bias [14], people see less of their own face in morphs with negatively valenced faces than in morphs with positively valenced faces, both in terms of attractiveness [15] and trustworthiness [16]. Enfacement itself is also correlated with the attractiveness of the other person’s face [9,10], and the strength of the enfacement illusion is stronger for positively perceived others [17]. These studies of social and emotional influences on visual self-face recognition and the enfacement illusion have largely focused on positive interpersonal dimensions (e.g., empathy, attractiveness, and trustworthiness). The effect of negative valence on the enfacement illusion has not been explored as thoroughly. Moreover, no published study to date has examined how varying the emotion expressed by another person during an interpersonal visuo-tactile experience might impact the degree of self/other merging.

Emotional facial expressions convey an intention to communicate, and may thus motivate greater effort on the part of the observer to understand the affective state of the other person. Fear is particularly important as a communicative signal because it usually indicates the presence of an immediate threat. Efficient and accurate recognition of a fearful facial expression confers both a private advantage to the observer by warning her of a potential threat to herself and a social benefit by facilitating an appropriate reaction to the distress of her companion. One might predict that enfacement would be especially susceptible to fearful facial expressions because they carry such valuable information for the adaptive behavior of the observer.

Perhaps because they are important for both personal safety and social interaction, seeing fearful faces produces greater resonance in somatosensory cortex than seeing other emotional expressions [18–20]. Somatosensory resonance is a process whereby the facial expressions of others are simulated at a sub-phenomenal level within one’s own sensorimotor systems to facilitate emotion recognition [21,22]. Evidence for a stronger somatosensory representation of fearful faces than other emotional expressions comes from a study in which transcranial magnetic stimulation (TMS) over the right somatosensory cortex impeded recognition of fearful but not happy facial expressions [18]. Another study found that seeing a fearful face being touched enhanced detection of near-threshold tactile stimulation on the observer’s own face compared to seeing neutral, happy, or angry faces being touched [19], suggesting that fearful faces enhance somatosensory activity more than other facial expressions, including similarly negative expressions like anger. Finally, a third study found that enfacement facilitated subsequent recognition of fear expressed by the assimilated face, but the illusion had no effect on recognition of happy or disgusted faces [20].

To summarize, an observer who sees a fearful face might be more motivated to understand the other’s affective state because of the value of the information that person is communicating. Achieving this understanding would entail simulating the other’s emotional state, thereby activating somatosensory cortex. During synchronous IMS, the somatosensory activation evoked by a fearful face, in combination with the somatosensory resonance induced by seeing another’s face touched in synchrony with one’s own [19,23,24], might increase enfacement beyond that obtained with neutral or emotional expressions of other kinds, including anger. Though angry expressions are similarly social and negative in valence, additional contextual information and knowledge of social contingencies are required to determine an appropriate reaction [25]. Moreover, the representation of anger in somatosensory cortex does not seem to be as strong as that of fear; whereas fearful facial expressions enhance remapping of seen touch onto the observer’s own somatosensory system, the same is not true for angry expressions [19]. Therefore, one might predict stronger enfacement of a fearful face than an angry or a neutral face.

The present experiment examined whether varying the emotional content of faces would affect the strength of the enfacement effect. In each experimental session, participants saw a fearful, an angry, or a neutral face being touched with a cotton swab either synchronously or asynchronously with a cotton swab touching their own face. Before and after this period of IMS, the participants watched a video of the same person’s face (with a neutral expression) gradually morphing into their own face, and stopped the morph video as soon as it began to look more like their own face than the other person’s face. We did not vary the emotional expression of the other person in the morph video because our hypothesis was based on a fear-specific enhancement of somatosensory resonance. Thus, the crucial time for presentation of the emotional expression was the period in which participants saw the other person being touched while feeling touch on their own face. The difference in the amount of the other person’s face in the frame where the participant stopped the morph video before and after synchronous IMS was taken as a measure of enfacement. The asynchronous IMS session controlled for any effects of exposure to uncorrelated visual and tactile stimulation, as well as any effect of mere familiarity with the other person’s face. A questionnaire was also used to assess the subjective strength of the enfacement illusion. It was predicted that enfacement would be comparable for neutral and angry faces, whereas seeing a fearful face would strengthen the enfacement effect due to enhanced somatosensory resonance and greater motivation to understand the other’s affective state. Instead, neither fearful nor angry expressions influenced the strength of the enfacement effect. This result is discussed in light of an asymmetric relationship between the processing of facial identity and facial expressions [26–30]

## Materials and Methods

### Participants

Fifty-four female volunteers between 19 and 32 years old were recruited from the University of Bologna and randomly assigned to one of three experimental groups, resulting in three groups of 18 participants each (Fear group: *M* = 25.06 years old, *SD* = ±1.83 years; Anger group: *M* = 25.06 years old, *SD* = ±2.21 years; Neutral group: *M* = 23.50 years old, *SD* = ±2.43 years). Participants in the Neutral group were only slightly younger on average than participants in the Fear group, *t*(34) = 2.17, *p* = .037, and the Anger group, *t*(34) = 2.01, *p* = .053. Each group saw a different emotional expression (fearful, angry, or neutral) during IMS. All participants had normal or corrected-to normal vision, reported normal tactile perception, and were naïve to the purpose of the experiment.

### Ethics

Participants gave written informed consent to participate in the study, which was approved by of the Ethics Committee for Psychological Research at the University of Bologna Department of Psychology. They were treated in accordance with the ethical standards of the 1964 Declaration of Helsinki.

### Materials

**Fig 1:**
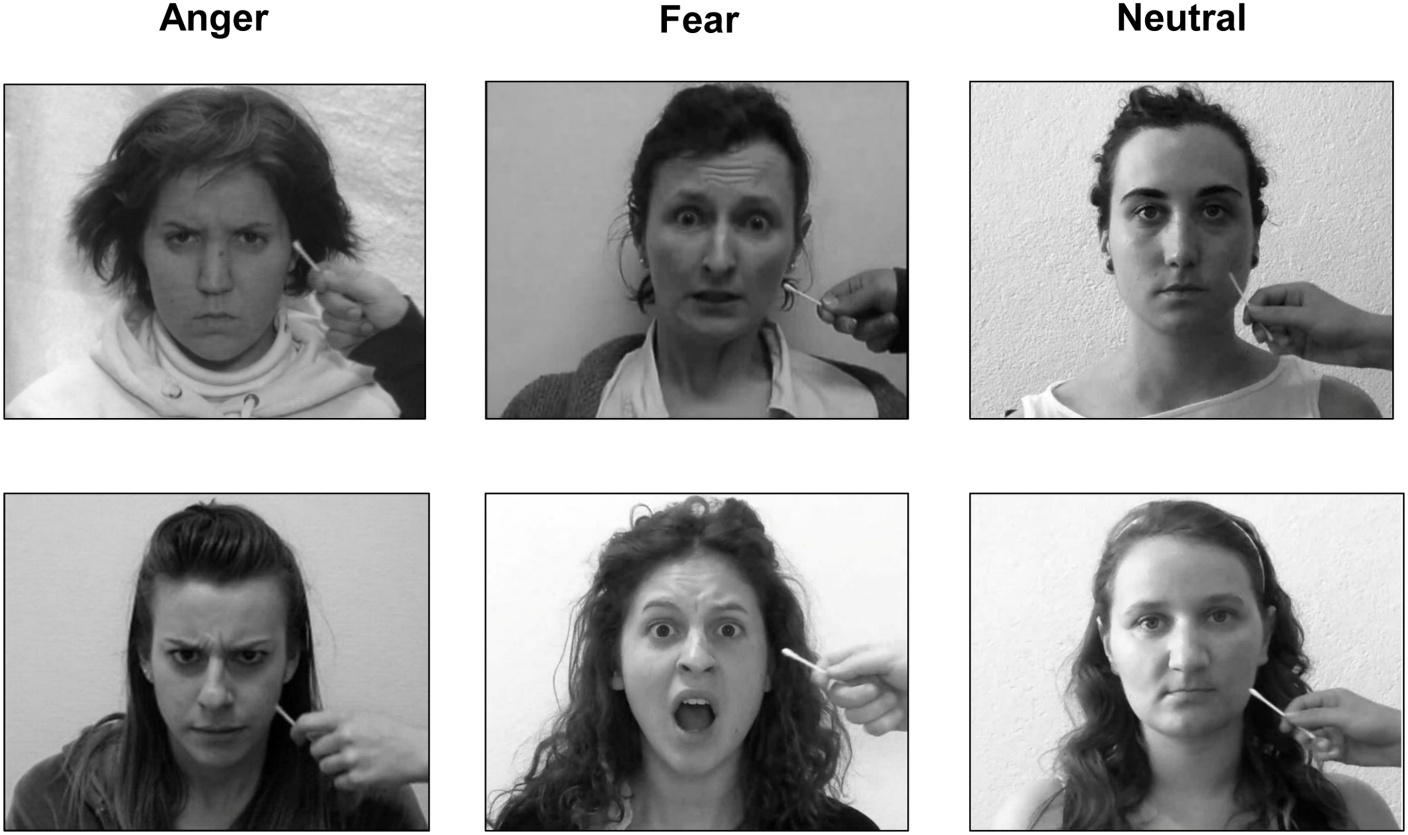
Frames from the angry, fearful, and neutral videos shown during IMS. Each participant saw videos from only one of the three facial expression categories. The assignment of each video to either the synchronous or the asynchronous IMS session was counterbalanced between participants. The individuals shown in this figure have given written informed consent (as outlined in the PLOS consent form) to have their likenesses published.

Prior to the testing session, a photograph of each participant's face with a neutral expression was taken with a digital camera. The photographs were converted to black-and-white, mirror-transposed, and overlaid with an oval template on a black background to remove hair and ears. Photographs of six adult female volunteers who did not participate in the experiment were obtained and processed in the same manner (except for the mirror-transposition) for use as the other-faces. Participant and other-face photographs were matched in luminance. These photographs were then blended in Abrosoft Fantamorph 4 to create dynamic morph videos that progressed from 100% other-face to 100% self-face. The morph videos were 100 s long and progressed at a rate of 1% change in face per second, resulting in a prolonged and subtle morph. Each participant’s face was morphed with two of the other-faces. Note that both the faces of the participants and the other-faces had neutral expressions in the morph videos. Additionally, a camcorder was used to record videos of the other-faces being stroked on the left cheek with a cotton swab. Each video was in full color and 120 s long with strokes occuring approximately every 2 s. While being touched, each volunteer maintained a facial expression (fearful, angry, or neutral) for approximately 10 s, and this segment was then looped to produce the full 120 s video (Fig 1). To make the neutral videos appear more natural, and to ensure that all three video types showed some kind of facial movement, we created the neutral videos from looped segments that included eye blinks, mild head movements, and mild facial muscle contractions.

The videos used for IMS were rated in a pilot study by a separate group of 25 female volunteers recruited from the same university as the participants in the main experiment (*M* = 24.64 years old, *SD* = ±9.59 years). These volunteers did not differ in age from the participants in the main experiment, *t*(77) = -0.08, *p* = .940. Volunteers in the pilot study categorized the emotional expressions in the videos (from the options of fear, happiness, surprise, disgust, anger, sadness, or neutral) and rated emotion intensity (from 1, “not at all,” to 7, “very much”). They also rated the attractiveness of the faces in the neutral photographs (from 1, “not at all,” to 7, “very much”) and how much the person in each video resembled their corresponding photograph (from 1, “not at all,” to 7, “it’s the same face”). (Please see S1 Supplementary Materials and S1 Table for the results of the pilot data analysis.)

Presentation 15.0 was used to display the morph and IMS videos and to collect participants’ responses to the morph videos. Additionally, after each stimulation session, participants completed a 14-item questionnaire to assess the strength of the subjective enfacement illusion experience. Table 1 contains the full text of this questionnaire, translated from Italian into English. Participants rated each statement on a scale of -3 (strongly disagree) to +3 (strongly agree), with 0 representing “neither agree nor disagree.”

**Table 1:** Text of questionnaire items used to assess participants’ subjective experience of the enfacement illusion.

Item	Item Text (translated from Italian)
01	While the other person's face was touched I seemed to feel the touch on my own face.
02	It seemed that the touch I felt on my face was caused by the cotton swab touching the other person's face.
03	It seemed that the other person's face was mine.
04	It seemed that the other person's face was part of my body.
05	It seemed that the other person's face belonged to me.
06	I seemed to see my face reflected in a mirror rather than the other person's face.
07	It seemed that the shape of the other person's face began to resemble mine.
08	It seemed that the skin color of the other face began to resemble mine.
09	It seemed that the features of the other person's face began to resemble mine.
10	It seemed that the other person's face would move if I moved.
11	I felt like I could control the other person's face.
12	I felt like I could not control my face.
13	I felt like I could not remember what my face looked like.
14	It seemed that sensation on my face was less vivid than normal.

### Procedure

**Fig 2:**
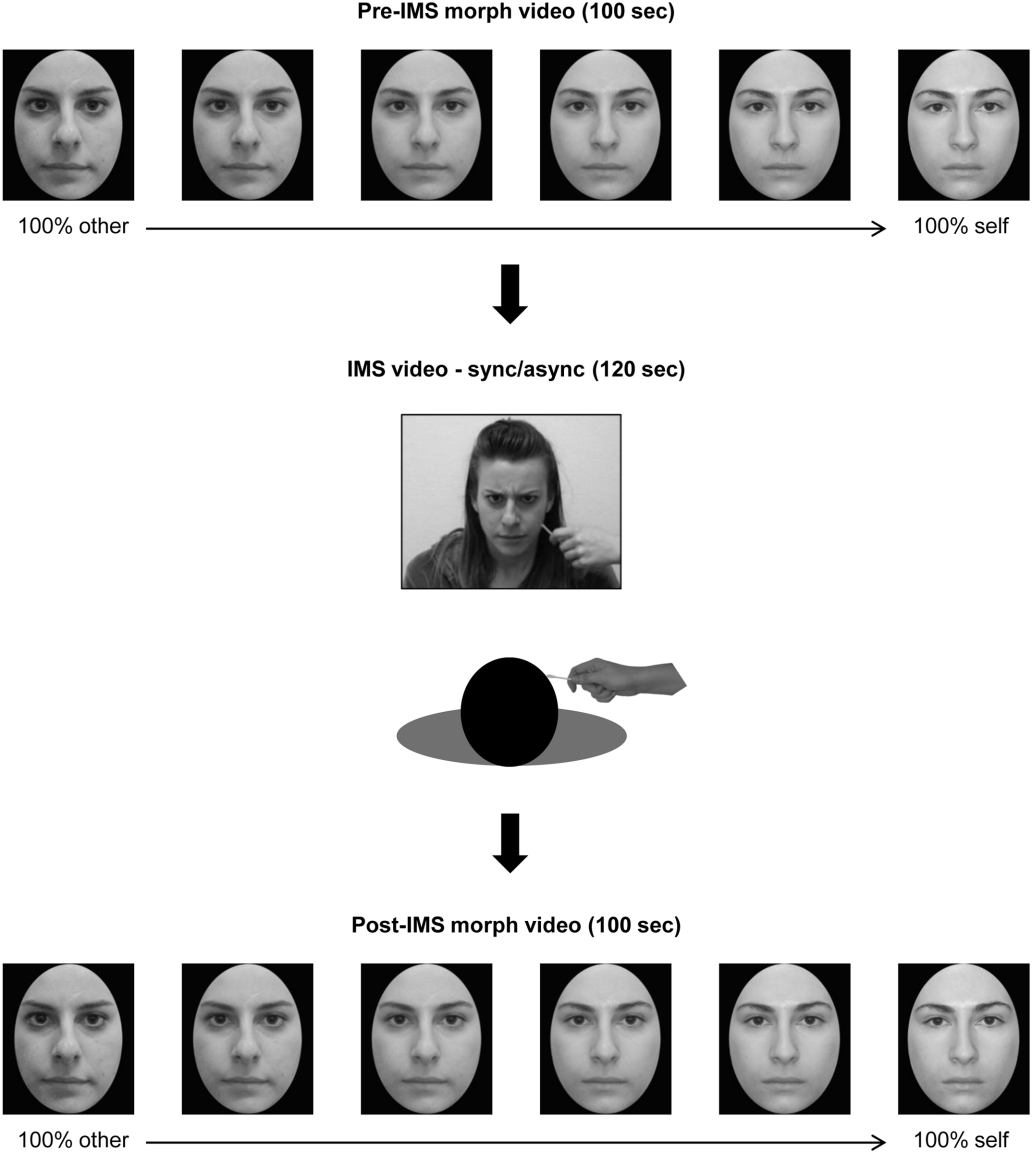
Diagram of an experimental session. Participants first watched an other-to-self morph video and pressed a button to stop it as soon as it began to look more like their face than the other person’s face. This was followed by a period of synchronous or asynchronous IMS, and then a repetition of the morph video post-IMS. Morph videos were black-and-white, but IMS videos were shown in full color. The individuals shown in this figure have given written informed consent (as outlined in the PLOS consent form) to have their likenesses published.

Participants completed one synchronous and one asynchronous IMS session, separated by at least 1 hour. Each participant saw only one type of facial expression in the IMS videos, either neutral, fearful, or angry. The order in which participants completed the IMS conditions was counterbalanced between participants within each group, as was the assignment of each other-face to either the synchronous or asynchronous IMS condition. A diagram of an experimental session is shown in Fig 2. In each session, participants first saw a morph video that changed from 100% other-face to 100% self-face. They were instructed to stop the video as soon as it began to look more like their own face than the other person’s face by pressing the “M” key. After this response, they watched a 120-s video of the other person continuously expressing either fear, anger, or a neutral expression while being stroked on the left cheek with a cotton swab. Concurrently, the participant was stroked on the right cheek (for specular correspondence) with a cotton swab either in synchrony or 1-s asynchrony with the touch in the video. Participants were instructed to sit still, to watch the face for the duration of the IMS video, and to attend to both the seen and the felt touch. Immediately after the IMS period, participants saw the same morph video as before and responded to it according to the same instructions. Finally, participants completed the illusion questionnaire at the end of each session. Questionnaire items were presented in a random order.

### Design and Analysis

This experiment employed a 2 x 2 x 3 mixed factors design, with time (pre- or post-IMS) and IMS mode (synchronous or asynchronous) as within-subjects variables and facial expression (fearful, angry, or neutral) as a between-subjects variable. The key dependent variable was the amount of the other’s face contained in the frame where participants stopped the morph video. Responses to the questionnaire items also served as a subjective measures of the enfacement illusion.

## Results

### Morph video analysis

A 2 x 2 x 3 mixed factors ANOVA with time (pre- or post-IMS) and IMS mode (synchronous or asynchronous) as within-subjects factors and the other’s facial expression during IMS (fearful, angry, or neutral) as a between-subjects factor was conducted on the amount of the other person’s face in the frame where participants stopped the morph video. This yielded a main effect of time, *F*(1, 51) = 10.96, *p* = .002, showing that, overall, participants stopped the morph videos sooner (at a frame containing less of their own face and more of the other person’s face) after IMS than before.

More importantly, there was an interaction between time and IMS mode, *F*(1, 51) = 8.96, *p* = .004. Paired samples t-tests were then used to make post-hoc comparisons. A Bonferroni correction was applied to control for multiple comparisons. The corrected significance level is *p* < .013. Participants stopped the morph video at a frame containing more of the other person’s face after synchronous IMS (*M* = 52.57%, *SE* = ±1.75%) than they did before synchronous IMS (*M* = 47.42%, *SE* = ±1.51%), *t*(53) = -4.92, *p* < .001, indicating that the experience of concurrent visual and tactile stimulation led them to accept more features of the other’s face as their own. Importantly, in sessions with asynchronous IMS, there was no difference in the amount of the other person’s face in the frame where participants stopped the morph video before (*M* = 49.11%, *SE* = ±1.71%) and after (*M* = 49.87%, *SE* = ±1.65%) IMS, *t*(53) = -0.61, *p* = .542, ruling out the possibility that mere exposure to the other’s face with incongruent visual and tactile input could produce the same effect. These results are consistent with previous enfacement studies [8,10]. Pre-IMS morph video stopping points did not differ between synchronous (*M* = 47.42%, *SE* = ±1.51%) and asynchronous (*M* = 49.11%, *SE* = ±1.71%) IMS sessions, *t*(53) = -0.91, *p* = .368. The difference between post-IMS morph video stopping points in the synchronous (*M* = 52.57%, *SE* = ±1.75%) and asynchronous (*M* = 49.87%, *SE* = ±1.65%) IMS sessions was also non-significant, *t*(53) = 1.55, *p* = .128. Though one might expect enfacement to be reflected in a difference between the frame that participants chose after synchronous and asynchronous IMS, the crucial comparisons are between the post-IMS morph video judgment and the corresponding pre-IMS baseline judgment made in a single experimental session.

**Fig 3:**
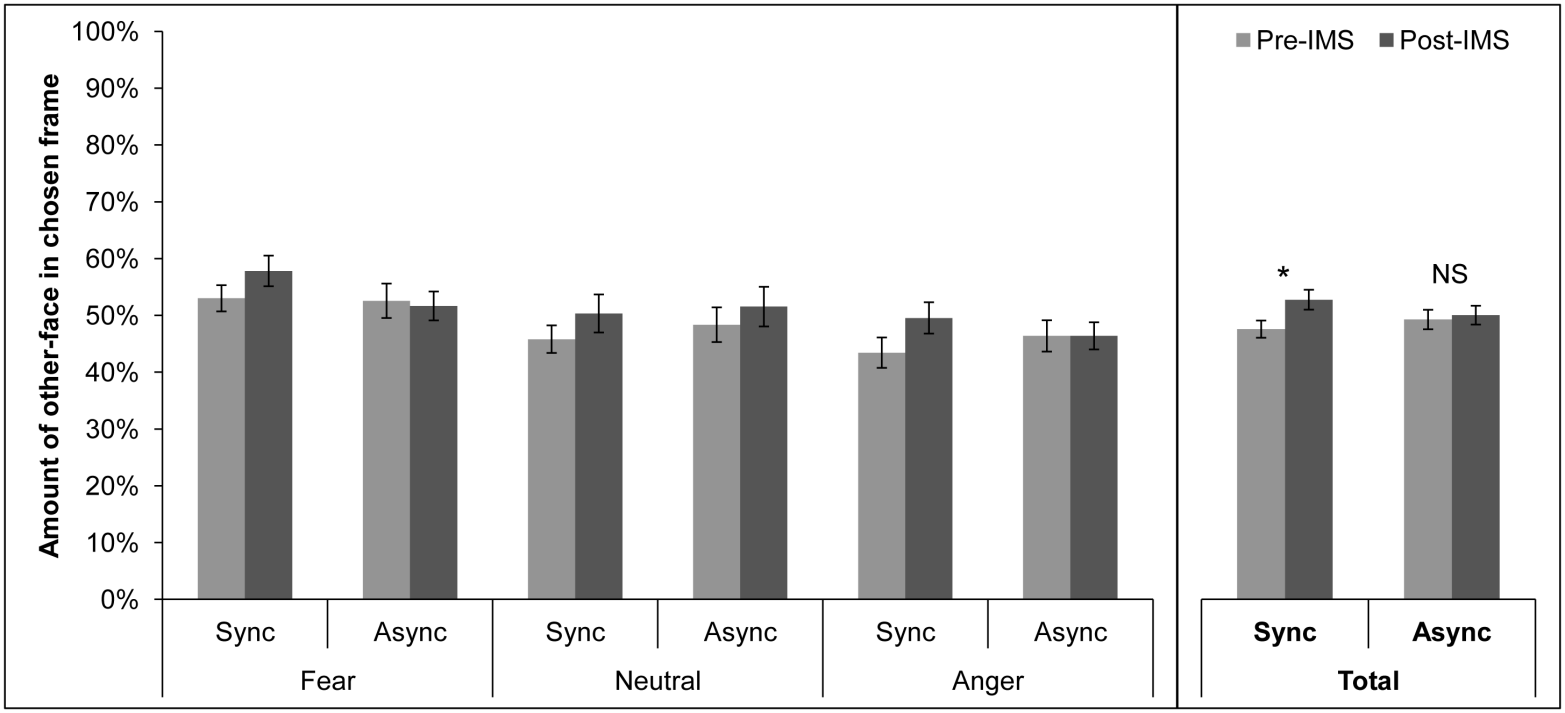
Mean percentage of other-face (±SEM) in the frame where participants stopped the morph video. Participants were instructed to stop the video as soon as the face began to look more like their own than the other person’s. Enfacement is evident when participants stop the video at a frame containing more of the other person’s face after synchronous IMS than before. * = sig. at *p* < .050; NS = non-sig.

Surprisingly, the other person’s facial expression during IMS had no impact on enfacement. There was no main effect of facial expression, *F*(2, 51) = 2.84, *p* = .068, no interaction between facial expression and time, *F*(2, 51) = 0.38, *p* = .685, or IMS mode, *F*(2, 51) = 0.84, *p* = .439, and, critically, no three-way interaction between emotion, time, and IMS, *F*(2, 51) = 1.09, *p* = .344. Contrary to our hypothesis, fearful faces did not increase enfacement relative to angry or neutral faces. In fact, the size of the enfacement effect was the same regardless of the emotion expressed by the other person during synchronous IMS (Fig 3).

To check whether differences in baseline, pre-IMS morph video judgments between groups might have obscured differences in the magnitude of enfacement between facial expression conditions, we performed a 2 (IMS mode) x 3 (facial expression) mixed factors ANOVA on the difference between the amount of the other person’s face in the frame participants chose before and after IMS. This approach mitigates the baseline differences between groups. The analysis revealed a significant main effect of IMS mode, *F*(1, 51) = 8.96, *p* = .004, with a larger pre/post-IMS difference in the synchronous IMS session (*M* = 5.15%, *SE* = ±1.05%) than in the asynchronous IMS session (*M* = 0.76%, *SE* = ±1.24%). There was neither a main effect of facial expression, *F*(2, 51) = 0.38, *p* = .685, nor an interaction between IMS mode and facial expression, *F*(2, 51) = 1.09, *p* = .344. These findings parallel our original analysis, indicating that any differences in baseline morph video judgments between groups did not obscure an effect of facial expression on the size of the enfacement illusion.

### Questionnaire analysis

Shapiro-Wilk tests revealed that enfacement questionnaire responses were not normally distributed, so non-parametric tests were used for all analyses of the questionnaire data. Due to a computer error, questionnaire responses were not collected after the synchronous IMS session for one participant, so analyses are based on the responses of the remaining 53 participants. Two other participants failed to provide a valid response to one of the questionnaire items (Item 01 in the asynchronous session in one case, and Item 09 in the synchronous session in the other), so analyses of these questionnaire items are based on the data from the remaining 52 participants.

**Fig 4:**
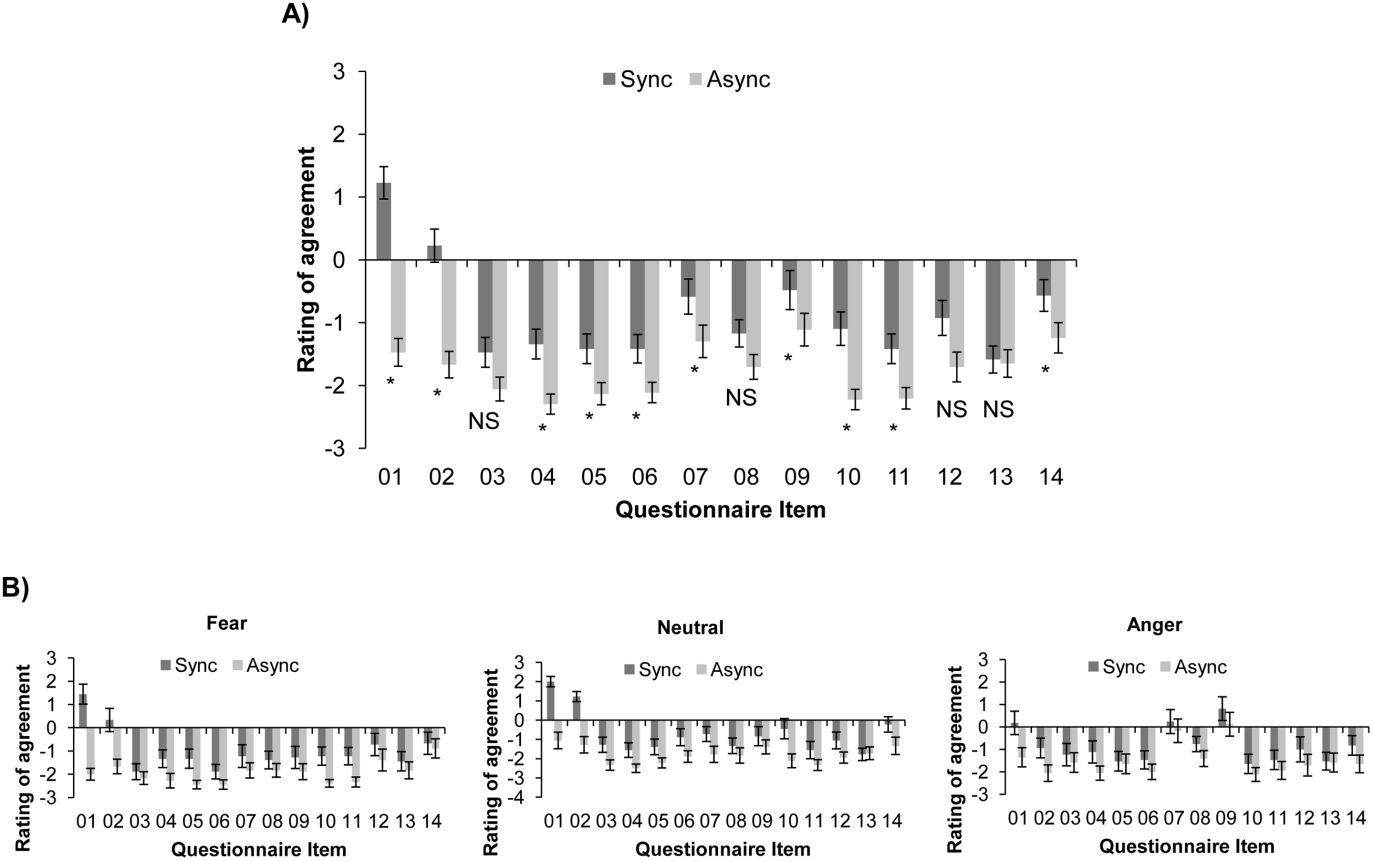
Mean ratings of agreement (±SEM) with enfacement illusion questionnaire items. Ratings were made on a Likert scale from -3 (strongly disagree) to 3 (strongly agree). Enfacement is evident in higher ratings of agreement after synchronous IMS than after asynchronous IMS. A) Mean ratings averaged across facial expression conditions. * = sig. at *p* < .004 (Bonferroni correction), one-tailed; NS = non-sig. B) Mean ratings in each facial expression condition. Note that there were no significant interactions between IMS mode and facial expression for any of the questionnaire items.

First, responses to each of the 14 questionnaire items were averaged across emotion conditions to look for a main effect of IMS mode (synchronous vs. asychronous), with higher ratings of agreement predicted in the synchronous session than in the asynchronous session. Ratings were compared using a series of one-tailed Wilcoxon signed-rank tests with a Bonferroni correction to control the family-wise error rate. Participants gave higher ratings of agreement to 10 of the 14 illusion questionnaire items after synchronous relative to asynchronous IMS (Fig 4), indicating that synchronous IMS successfully induced a subjective illusion of self/other merging.

To test for an interaction between IMS mode and emotional expression, we calculated the differences between participants’ ratings of each questionnaire item in the synchronous and asynchronous sessions (synchronous rating—asynchronous rating). These difference scores reflect the amount of enfacement that each participant experienced. We then used Bonferroni-corrected Kruskal-Wallis tests to look for differences in the strength of the enfacement illusion between the groups that viewed fearful, angry, and neutral faces. No differences between emotion conditions were significant, again showing that enfacement was not influenced by the other person’s facial expression.

## Discussion

In the enfacement paradigm, synchronous interpersonal visual and tactile inputs update the self-face representation, causing the other person’s face to be assimilated. We hypothesized that seeing a fearful face being touched in synchrony with one’s own face would increase enfacement because of greater motivation to understand the affective state of the other person, a process that would involve enhanced somatosensory resonance. We also predicted that seeing an angry face being touched in synchrony with one’s own face would *not* increase enfacement because anger is a more complex social emotion that requires additional contextual information to be properly understood [25], and because angry expressions do not appear to be as strongly represented in the somatosensory system of the observer [19]. Contrary to our hypotheses, the strength of enfacement was the same regardless of the emotion expressed by the other person. Neither fearful nor angry facial expressions affected the amount of self/other merging produced by synchronous IMS.

Previous studies have shown that visual self-face recognition from self/other morphs is influenced by the affective valence of the other person’s face, especially in terms of positive interpersonal dimensions such as attractiveness and trustworthiness [15,16]. In contrast, the present study did not find an effect of the other person’s emotional expression on self-face recognition from other-to-self morphs. It is important to remember, however, that our morph videos always showed both the participant’s face and the other person’s face with *neutral* expressions. The emotion manipulation only occurred in the IMS video. Our intention was not to show a direct influence of the emotional content of the self/other morph on self-face recognition, but to investigate whether seeing a fearful face would enhance somatosensory resonance, thereby strengthening the enfacement illusion induced by synchronous IMS. Instead, we found that varying the other person’s facial expression did not influence the strength of enfacement. Moreover, these earlier studies found an effect of positive interpersonal dimensions on self-face recognition from self/other morphs, whereas the present study investigated the effect of negative emotional expressions on the synchronous IMS procedure that induces enfacement.

Responses to the enfacement questionnaire further confirmed that participants experienced an illusion of enfacement, but this effect was not influenced by the other person’s facial expression. Participants gave higher ratings of agreement to 10 of the 14 questionnaire items after synchronous IMS compared to asynchronous IMS, including statements assessing both self-identification (Items 01, 02, 04, 05, 06, 10, and 11) and physical similarity (Items 07, 09, and 14) [11]. Moreover, the differences between the ratings given to these questionnaire items in synchronous and asynchronous sessions did not change according to the emotion expressed during IMS. This suggests that the subjective experience of enfacement with emotional faces is both quantitatively and qualitatively similar to enfacement with neutral faces.

Cardini and colleagues [19] found that viewing fearful faces being touched improves detection of near-threshold tactile stimuli on one’s own face. The present study, on the other hand, did not find a fear-specific enhancement of enfacement. Although both of these experiments presented tactile stimuli on the participant’s face while the participant viewed touch on another person’s face, they were actually measuring quite different things. Cardini and colleagues looked at the effect of congruent interpersonal visuo-tactile stimulation on a concurrent tactile detection task, which is directly related to activity in somatosensory cortex. Instead, our study measured *visual* self-face recognition *after* inducing an illusion with synchronous IMS. Modulation of somatosensory cortical activity by fearful facial expressions might not be expected to have as clear an effect in this paradigm as it does in a tactile detection paradigm.

Additionally, Maister and colleagues [20] demonstrated that inducing an enfacement illusion enhances detection of fear when it is expressed by the person whose face was assimilated. As discussed earlier, somatosensory simulation seems to be particularly important for recognizing fearful facial expressions [18], so Maister and colleagues reasoned that synchronous IMS improved fear detection by enhancing resonance with somatosensory events on the other person’s face. Nevertheless, the results of the present study suggest that this effect is not reciprocal. Viewing fearful facial expressions did not strengthen assimilation of the other person’s face via synchronous IMS. This suggests that there is an asymmetric relationship between the multisensory interactions underlying enfacement and the processing of facial expressions.

A long-standing view of face perception holds that facial identity and facial expressions are processed independently [31,32], allowing one to recognize individuals from their faces regardless of changes in expression. This view is supported by case studies of patients with impaired facial identity recognition but spared facial expression recognition [33–36] and evidence for segregated processing of facial identity and facial expressions in healthy participants [37–40]. Though more recent reports have challenged the idea of completely independent processing streams for facial identity and expressions, they still tend to support some degree of independence between the two functions (see [41] for a review). In fact, several studies have shown that facial identity influences facial expression processing, but not vice versa [26–30]. One could see how this asymmetric relationship between facial identity and expression processing might be benefical. The identity of a familiar person provides additional information relevant to interpreting emotional expressions, such as knowledge of the individual’s personality and prior interactions with them. On the other hand, it could be detrimental to identity recognition if dynamic facial attributes such as emotional expressions were to affect facial identity processing. The results of the present study, together with the results of Maister and colleagues [20], support an asymmetric relationship between facial identity and facial expression processing and extend it into the domain of dynamic self-face recognition processes. While assimilation of another person’s face into the self-face representation can affect perception of that person’s facial expressions [20], the expression displayed by another person’s face does not affect how readily that face is assimilated.

The asymmetric relationship between facial identity and facial expression processing may also shed light on why fearful expressions did not affect enfacement, in spite of their significance for adaptive behavior. Fearful expressions usually signal the presence of an immediate threat in the observer’s environment. Recognizing and reacting appropriately to fearful faces would therefore be critical for personal survival. Though this might make fearful faces especially salient, it does not necessarily follow that they should impact the process of visual self-face recognition, or even facial identity processing in general. As mentioned earlier, facial identity recognition might be hindered if it were easily influenced by dynamic facial expressions.

Though the present study did not find an effect of fearful or angry facial expressions on enfacement, it does not rule out the possibility that other facial expressions might modulate the illusion. In contrast to the asymmetric interaction model of facial identity and facial expression processing, some studies have found that happy facial expressions can facilitate the identification of faces as familiar [42–44]. However, another study found that happy facial expressions increased the familiarity ratings of both familiar and unfamilliar faces [45]. This suggests that happy facial expressions might just bias the perceiver towards a feeling of familiarity rather than enhancing facial identity recognition per se. A later study demonstrated that happy facial expressions can also facilitate explicit recognition (i.e., naming) of famous faces [46]. Though the authors of that study argued that an internet search yielded at least as many photographs of their famous personalities with neutral expressions as with happy expressions, it is difficult to rule out the possibility that the facilitatory effect they found could be due to greater exposure to the smiling expressions of those celebrities. Despite the methodological issues with these studies, a future study could try the enfacement paradigm with happy facial expressions to determine whether smiling faces might affect the dynamic processes underlying self-face identification in a way that fearful and angry faces do not.

One might argue that visuo-motor congruence was not balanced across the three facial expression conditions in this experiment. Although even the neutral IMS videos included mild facial movements such as eye blinks that would likely have been incompatible with the participant’s own, these movements are not as sustained as those produced in the emotional facial expression videos. Studies on similar embodiment illusions typically induced by visuo-tactile synchrony have shown that visuo-motor congruence can also impact the assimilation of external bodies or body parts [47–49]. Thus, we cannot exclude the possibility that the discrepancy between the facial movements of the actors in the fearful and angry videos and the participants’ own facial muscles may have reduced the enfacement illusion, irrespective of any emotional component per se. This could potentially have obscured an emotion-related enhancement of enfacement. Future studies could attempt to test this possibility by controlling visuo-motor congruence independently of emotional expression.

Another potential limitation of our study is that we did not measure participants’ empathic traits, so we cannot exclude the possibility that the groups may have differed in this respect. A previous study found that people with higher levels of both cognitive and emotional components of empathy tend to be more susceptible to the enfacement illusion [10]. Empathy may also influence reactions to emotional stimuli. For instance, participants high in emotional empathy show more automatic mimcry of happy and angry facial expressions [50]. Additionally, cognitive empathy tends to primarily influence reactions to positive emotional stimuli, whereas emotional empathy influences reactions to negative emotional stimuli [51]. It is thus possible that more empathic individuals might show differential enfacement effects depending on the other’s facial expression. Future studies could explore the relationship between participant empathy and emotional expression in determining the strength of enfacement.

In conclusion, the results of the present study suggest that negative emotional facial expressions do not modulate the extent to which another’s face is assimilated into the self-face representation following synchronous interpersonal visuo-tactile stimulation. Together with the results from a previous study showing that enfacement can enhance fear recognition [20], our results support an asymmetric interaction between facial expression processing and dynamic, multisensory processes of self-face identification. Further research is needed to determine whether this asymmetric interaction holds for all facial expression processing or is specific to negative emotional expressions.

## Supporting Information

S1 Dataset Pilot and experimental data. (XLSX)

S1 Supplementary Materials Pilot data analysis. (DOCX)

S1 Table Pilot ratings of the IMS videos showing fearful, angry, and neutral expressions, and the corresponding photographs showing all the actors with neutral expressions. (DOCX)
