# Supplementary material for: The Enfacement Illusion Is Not Affected by Negative Facial Expressions
Source: PLoS One. 2015 Aug 20;10(8):e0136273. doi: 10.1371/journal.pone.0136273 (PMC4546364; doi:10.1371/journal.pone.0136273)
Supplement: S1 Supplementary Materials — (DOCX) [file pone.0136273.s002.docx]

**S1 Supplementary Materials**

The pilot ratings of the IMS videos and the corresponding neutral face photographs of the same individuals used for the morph videos are shown in S2 Table. Non-parametric tests were used for all analyses because the data were not normally distributed. A Cochran’s Q test showed that the percentage of participants who correctly classified the emotion expressed in each IMS video did not differ between the videos, *Χ^2^*(5) = 10.46, *p* = .067.

Emotion intensity, attractiveness, and likeness-to-photograph ratings were analyzed with Friedman’s ANOVAs. Note that emotion intensity was rated from the IMS videos, whereas attractiveness ratings were based on the neutral photographs of the actors used in the morph videos. Likeness-to-photograph ratings were made by comparing the IMS video of each actor to her neutral photograph. The facial expressions in the six IMS videos were perceived as equally intense, *Χ^2^*(5) = 10.52, *p* = .062. However, the photographs of the neutral faces differed in attractiveness, *Χ^2^*(5) = 37.91, *p* < .001. Post-hoc Wilcoxon signed-rank tests with a Bonferroni correction for multiple comparisons showed that the neutral photographs corresponding to Neutral Video 1 and Anger Video 1 were perceived as more attractive than the neutral photographs corresponding to Neutral Video 2, Anger Video 2, and Fear Video 1 (*p* < .003, uncorrected, in all cases). To determine whether this might complicate interpretation of the data from the main experiment, we conducted another Friedman’s ANOVA on the average attractiveness ratings of the neutral photographs associated with each IMS video category. This test was not significant, *Χ^2^*(2) = 3.43, *p* = .184, indicating that average attractiveness was well balanced across IMS emotion categories. It is not a problem that the neutral and anger IMS categories were each associated with one neutral face that was perceived as more attractive because the assignment of each other-face to the synchronous or the asynchronous IMS session was counterbalanced across participants.

The IMS videos also differed in likeness to their corresponding neutral photograph, *Χ^2^*(5) = 23.76, *p* < .001. Wilcoxon signed-rank tests with a Bonferroni correction showed that Anger Video 2 was perceived as more similar to its corresponding neutral photograph than Fear Video 1, Fear Video 2, Neutral Video 1, and Anger Video 1 (*p* < .003 in all cases). The concern here would be that enfacement might be more easily detected when participants experience synchronous IMS with Angry Video 2 because it is more obvious that the other-face in the morph video is the same as the face in the IMS video. To determine whether this was the case, we performed a post-hoc independent samples t-test on the differences between when participants stopped the morph video before and after synchronous IMS with Anger Videos 1 and 2, because any difference in enfacement between these two conditions would not be due to a difference in the type of emotional expression. The amount of enfacement did not differ depending on which angry face participants saw being touched in synchrony with their own, *t*(16) = 0.016, *p* = .987. Thus, the higher likeness-to-photo rating given to Anger Video 1 did not bias the design by enhancing the ability of the morph video task to detect an enfacement effect when that stimulus was used.
