# Supplementary material for: The Enfacement Illusion Is Not Affected by Negative Facial Expressions
Source: PLoS One. 2015 Aug 20;10(8):e0136273. doi: 10.1371/journal.pone.0136273 (PMC4546364; doi:10.1371/journal.pone.0136273)
Supplement: S1 Table — (DOCX) [file pone.0136273.s003.docx]

**S2 Table. Pilot ratings of the IMS videos showing fearful, angry, and neutral expressions, and the corresponding photographs showing all the actors with neutral expressions.**

| **Other-Face^a^** | **Emotion Recognition^b^** | **Emotion**  **Intensity^c^** | **Photo Attractiveness^c^** | **Likeness**  **to Photo^c^** |
| --- | --- | --- | --- | --- |
| Fear 1 | 92% | 5.8 | 2.8 | 4.2 |
| Fear 2 | 80% | 5.5 | 3.7 | 3.8 |
| Anger 1 | 76% | 5.0 | 4.2 | 3.9 |
| Anger 2 | 96% | 5.6 | 2.8 | 6.0 |
| Neutral 1 | 88% | 5.7 | 4.2 | 4.3 |
| Neutral 2 | 100% | 5.9 | 3.1 | 5.0 |

^a^Each row refers to the IMS video showing an emotional expression, as well as the corresponding neutral photograph of the same person used for the morph videos.

^b^Volunteers chose between 7 emotion categories (fear, happiness, surprise, disgust, anger, sadness, or neutral).

^c^IMS video emotion intensity, photo attractiveness, and similarity of the face in the video to the face in the photo were rated on a scale of 1 (“not at all”) to 7 (“very much”).
